# (*E*,*E*)-Methyl 2-[(3-nitrobenzylidene)­aminomethyl]-3-phenylpropenoate

**DOI:** 10.1107/S1600536808043262

**Published:** 2008-12-24

**Authors:** Adailton J. Bortoluzzi, Tula B. Bisol, Marcus M. Sá

**Affiliations:** a Depto. de Química - UFSC, 88040-900 - Florianópolis, SC, Brazil

## Abstract

The mol­ecule of the title compound, C_18_H_16_N_2_O_4_, adopts a T-shaped conformation with *E* stereochemistry for the imine double bond. The (3-nitro­benzyl­idene)amino fragment is almost planar, the mean planes of phenyl ring and nitro group forming a dihedral angle of 8.9 (3)°. In the 3-phenyl­acryloyl unit, the acrylic ester fragment is also almost planar, with the phenyl ring twisted by 41.44 (7)°. In the crystal, the mol­ecules are linked by C—H⋯O hydrogen-bond inter­actions into chains running parallel to [01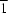].

## Related literature

For general background to the chemistry of Morita–Baylis–Hillman adducts, see: Singh & Batra (2008 ▶); Masson *et al.* (2007 ▶); Basavaiah *et al.* (2003 ▶). For background to this study, see: Bortoluzzi *et al.* (2006 ▶); Fernandes *et al.* (2004 ▶); Sá *et al.* (2006 ▶, 2007 ▶, 2008 ▶). For the synthesis, see: Sá (2003 ▶). For a description of the Cambridge Structural Database, see: Allen (2002 ▶); and of *MOGUL*, see: Bruno *et al.* (2004 ▶).
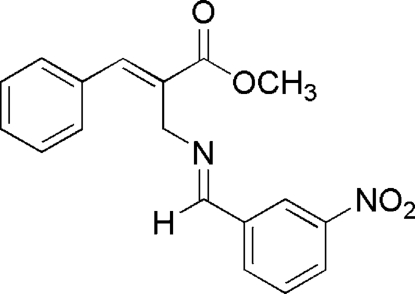


## Experimental

### 

#### Crystal data

C_18_H_16_N_2_O_4_
*M* *_r_* = 324.33
Triclinic, 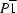

*a* = 8.6035 (12) Å
*b* = 8.7829 (14) Å
*c* = 12.4680 (14) Å
α = 79.275 (18)°
β = 76.526 (13)°
γ = 63.158 (14)°
*V* = 813.9 (2) Å^3^
*Z* = 2
Mo *K*α radiation
μ = 0.10 mm^−1^
*T* = 293 (2) K
0.50 × 0.30 × 0.20 mm

#### Data collection

Enraf–Nonius CAD-4 diffractometer
Absorption correction: none
3026 measured reflections
2883 independent reflections
2165 reflections with > 2σ (*I*)
*R*_int_ = 0.024
3 standard reflections every 200 reflections intensity decay: 1%

#### Refinement

*R*[*F* ^2^ > 2σ(*F* ^2^)] = 0.043
*wR*(*F* ^2^) = 0.124
*S* = 1.09
2883 reflections
218 parameters
H-atom parameters constrained
Δρ_max_ = 0.17 e Å^−3^
Δρ_min_ = −0.22 e Å^−3^

### 

Data collection: *CAD-4 Software* (Enraf–Nonius, 1989 ▶); cell refinement: *SET4* in *CAD-4 Software*; data reduction: *HELENA* (Spek, 1996 ▶); program(s) used to solve structure: *SIR97* (Altomare *et al.*, 1999 ▶); program(s) used to refine structure: *SHELXL97* (Sheldrick, 2008 ▶); molecular graphics: *PLATON* (Spek, 2003 ▶); *Mercury* (Macrae *et al.*, 2006 ▶); software used to prepare material for publication: *SHELXL97*.

## Supplementary Material

Crystal structure: contains datablocks global, I. DOI: 10.1107/S1600536808043262/rz2278sup1.cif

Structure factors: contains datablocks I. DOI: 10.1107/S1600536808043262/rz2278Isup2.hkl

Additional supplementary materials:  crystallographic information; 3D view; checkCIF report

## Figures and Tables

**Table 1:** Hydrogen-bond geometry (Å, °)

*D*—H⋯*A*	*D*—H	H⋯*A*	*D*⋯*A*	*D*—H⋯*A*
C23—H23⋯O15^i^	0.93	2.55	3.307 (3)	139

Symmetry code: (i) 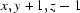.

**Fig. 1.**
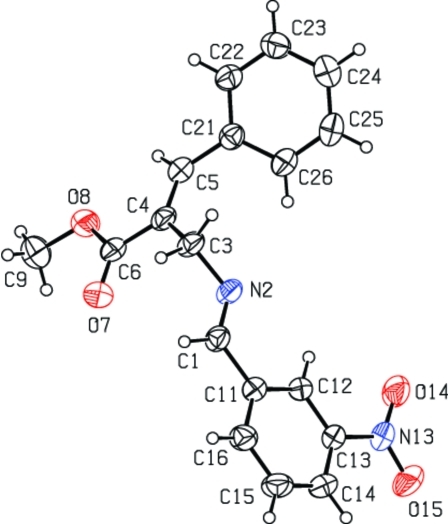
The molecular structure of the title compound with the labeling scheme. Displacement ellipsoids are shown at the 40% probability level.

**Fig. 2.**
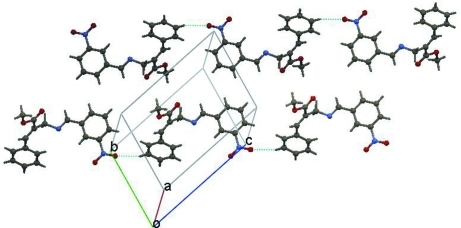
Packing diagram of the title compound showing the formation of chains parallel to the [0 1 1] direction. Hydrogen bonds are shown as dashed lines.

**Fig. 3.**
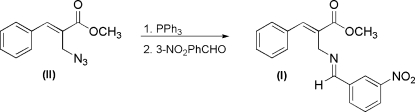
Reaction scheme.

## supplementary crystallographic information

### Comment

Nitrogen-containing building blocks derived from α-methylene-β-hydroxy esters (Morita-Baylis-Hillman adducts) have been widely employed in modern organic chemistry for the synthesis of natural products and heterocycles of biological relevance (Singh & Batra, 2008; Masson *et al.*, 2007; Basavaiah *et al.*, 2003). During our studies on the Morita-Baylis-Hillman reaction (Bortoluzzi *et al.*, 2006; Fernandes *et al.*, 2004; Sá *et al.*, 2006; Sá *et al.*, 2007; Sá *et al.*, 2008), we reported the high-yield preparation of *N*-allylic imine (I) (Scheme 1) and analogs by a tandem Staudinger/Aza-Wittig process involving allyl azide (II) and a combination of triphenylphosphine and 3-nitrobenzaldehyde (Sá, 2003). In spite of the full chemical characterization described for the multifunctional compound (I), the stereochemistry of the imine double bond could not be unambiguously elucidated and was tentatively assigned as being E on the basis of the available data (Sá, 2003).

The molecular structure of the title compound adopts a T-shaped conformation (Fig. 1). The E stereochemistry for the imine double bond was unambiguously elucidated by X-ray analysis. The torsion angles N2—C1—C11—C12 of -8.2 (3)° and C12—C13—N13—O14 of 9.2 (3)° demonstrate that the (3-nitrobenzylidene)amino moiety is almost planar. The plane of the nitro group deviates of 8.9 (3)° with respect to the mean plane of the parent phenyl ring. In the 3-phenylacryloyl moiety, the acrylic ester fragment is also almost planar with as indicated by the C5–C4–C6–O7 torsion angle of 175.67 (18)°, whereas the phenyl ring is twisted by 41.44 (7)°, probably due to steric effect. This observation indicates that the vinyl C═C double bond is strongly conjugated with the carboxyl group instead that with the aromatic phenyl ring. A search using *Mogul* (Bruno *et al.*, 2004) based on the CCDC system (version 5.29; Allen, 2002) revealed that the bond lengths found in the structure of (I) are within the expected range for organic compounds. In the crystal packing, molecules are linked by intermolecular C—H···O hydrogen bonding interactions (Table 1) to form chains running parallel to the [0 1 1] direction (Fig. 2).

### Experimental

Allyl imine (I) was prepared as previously described (Sá, 2003). Allyl azide (II) and triphenylphosphine (1.0 mmol each) were stirred in anhydrous CHCl_3_ (3.0 ml) and after evolution of N_2_ has ceased, 3-nitrobenzaldehyde (1.0 mmol) was added and the mixture was stirred for further 20 h at room temperature. Concentration of the final mixture and separation of triphenylphosphine oxide by crystallization from ethyl ether furnished a white solid (84% yield). A careful recrystallization from ethyl acetate/hexane (1:3 v/v) provided crystals (mp 114.3–115.3 °C) suitable for X-ray crystallographic analysis.

### Refinement

All H atoms were placed at idealized positions and refined as riding, with C—H = 0.93–0.97 Å and with *U*_iso_(H) = 1.2 *U*_eq_(C) or 1.5 *U*_eq_(C) for methyl H atoms.

### Crystal data

**Table:**

C_18_H_16_N_2_O_4_	*Z* = 2
*M**_r_* = 324.33	*F*(000) = 340
Triclinic, *P*1	*D*_x_ = 1.323 Mg m^−^^3^
Hall symbol: -P 1	Mo *K*α radiation, λ = 0.71069 Å
*a* = 8.6035 (12) Å	Cell parameters from 25 reflections
*b* = 8.7829 (14) Å	θ = 5.3–16.7°
*c* = 12.4680 (14) Å	µ = 0.10 mm^−^^1^
α = 79.275 (18)°	*T* = 293 K
β = 76.526 (13)°	Irregular block, colorless
γ = 63.158 (14)°	0.50 × 0.30 × 0.20 mm
*V* = 813.9 (2) Å^3^	

### Data collection

**Table:**

Enraf–Nonius CAD-4 diffractometer	*R*_int_ = 0.024
Radiation source: fine-focus sealed tube	θ_max_ = 25.1°, θ_min_ = 1.7°
graphite	*h* = −10→9
ω–2θ scans	*k* = −10→10
3026 measured reflections	*l* = −14→0
2883 independent reflections	3 standard reflections every 200 reflections
2165 reflections with > 2σ (*I*)	intensity decay: 1%

### Refinement

**Table:**

Refinement on *F*^2^	Primary atom site location: structure-invariant direct methods
Least-squares matrix: full	Secondary atom site location: difference Fourier map
*R*[*F*^2^ > 2σ(*F*^2^)] = 0.043	Hydrogen site location: inferred from neighbouring sites
*wR*(*F*^2^) = 0.124	H-atom parameters constrained
*S* = 1.09	*w* = 1/[σ^2^(*F*_o_^2^) + (0.0588*P*)^2^ + 0.1527*P*] where *P* = (*F*_o_^2^ + 2*F*_c_^2^)/3
2883 reflections	(Δ/σ)_max_ < 0.001
218 parameters	Δρ_max_ = 0.17 e Å^−^^3^
0 restraints	Δρ_min_ = −0.22 e Å^−^^3^

### Fractional atomic coordinates and isotropic or equivalent isotropic displacement parameters (Å^2^)

**Table:**

	*x*	*y*	*z*	*U*_iso_*/*U*_eq_	
C1	0.1755 (2)	0.5606 (2)	0.77725 (14)	0.0470 (4)	
H1	0.1648	0.6615	0.7983	0.056*	
N2	0.1722 (2)	0.55429 (18)	0.67793 (12)	0.0492 (4)	
C3	0.1642 (3)	0.7065 (2)	0.60179 (15)	0.0514 (5)	
H3A	0.2700	0.6741	0.5459	0.062*	
H3B	0.1627	0.7908	0.6430	0.062*	
C4	0.0043 (2)	0.7874 (2)	0.54518 (14)	0.0465 (4)	
C5	0.0074 (2)	0.7661 (2)	0.44100 (14)	0.0488 (4)	
H5	−0.0960	0.8330	0.4121	0.059*	
C6	−0.1594 (3)	0.9030 (2)	0.61246 (15)	0.0515 (5)	
O7	−0.1704 (2)	0.91839 (19)	0.70815 (11)	0.0734 (4)	
O8	−0.29582 (18)	0.98968 (18)	0.55747 (11)	0.0639 (4)	
C9	−0.4579 (3)	1.1040 (3)	0.6204 (2)	0.0796 (7)	
H9A	−0.4923	1.0411	0.6855	0.119*	
H9B	−0.5493	1.1532	0.5758	0.119*	
H9C	−0.4398	1.1936	0.6416	0.119*	
C11	0.1958 (2)	0.4135 (2)	0.86183 (14)	0.0446 (4)	
C12	0.1900 (2)	0.2681 (2)	0.83818 (13)	0.0434 (4)	
H12	0.1726	0.2610	0.7687	0.052*	
C13	0.2104 (2)	0.1340 (2)	0.91989 (14)	0.0471 (4)	
N13	0.2048 (2)	−0.01950 (19)	0.89347 (14)	0.0573 (4)	
O14	0.2052 (2)	−0.03191 (17)	0.79774 (13)	0.0745 (5)	
O15	0.2022 (3)	−0.13071 (19)	0.96882 (14)	0.0879 (5)	
C14	0.2357 (3)	0.1386 (3)	1.02445 (15)	0.0579 (5)	
H14	0.2487	0.0465	1.0780	0.069*	
C15	0.2412 (3)	0.2832 (3)	1.04694 (16)	0.0625 (5)	
H15	0.2582	0.2896	1.1166	0.075*	
C16	0.2215 (3)	0.4193 (3)	0.96652 (15)	0.0555 (5)	
H16	0.2255	0.5163	0.9829	0.067*	
C21	0.1565 (2)	0.6482 (2)	0.36716 (14)	0.0465 (4)	
C22	0.1946 (3)	0.7024 (2)	0.25635 (15)	0.0546 (5)	
H22	0.1252	0.8133	0.2294	0.066*	
C23	0.3330 (3)	0.5951 (3)	0.18584 (17)	0.0615 (5)	
H23	0.3578	0.6345	0.1122	0.074*	
C24	0.4353 (3)	0.4296 (3)	0.22366 (18)	0.0631 (5)	
H24	0.5284	0.3567	0.1758	0.076*	
C25	0.3986 (3)	0.3727 (3)	0.33283 (19)	0.0660 (6)	
H25	0.4671	0.2607	0.3585	0.079*	
C26	0.2610 (3)	0.4802 (2)	0.40479 (17)	0.0575 (5)	
H26	0.2379	0.4405	0.4785	0.069*	

### Atomic displacement parameters (Å^2^)

**Table:**

	*U*^11^	*U*^22^	*U*^33^	*U*^12^	*U*^13^	*U*^23^
C1	0.0563 (11)	0.0396 (9)	0.0488 (10)	−0.0228 (8)	−0.0137 (8)	0.0000 (7)
N2	0.0628 (9)	0.0407 (8)	0.0475 (9)	−0.0251 (7)	−0.0172 (7)	0.0061 (6)
C3	0.0672 (12)	0.0433 (10)	0.0499 (10)	−0.0310 (9)	−0.0165 (9)	0.0089 (8)
C4	0.0590 (11)	0.0372 (9)	0.0456 (10)	−0.0253 (8)	−0.0114 (8)	0.0066 (7)
C5	0.0529 (10)	0.0424 (9)	0.0495 (10)	−0.0203 (8)	−0.0123 (8)	0.0039 (7)
C6	0.0668 (12)	0.0407 (9)	0.0452 (10)	−0.0263 (9)	−0.0070 (9)	0.0059 (8)
O7	0.0964 (11)	0.0643 (9)	0.0464 (8)	−0.0243 (8)	−0.0094 (7)	−0.0052 (7)
O8	0.0579 (8)	0.0640 (9)	0.0542 (8)	−0.0156 (7)	−0.0047 (6)	−0.0037 (6)
C9	0.0652 (14)	0.0729 (15)	0.0783 (16)	−0.0156 (12)	0.0041 (11)	−0.0130 (12)
C11	0.0488 (10)	0.0412 (9)	0.0426 (9)	−0.0192 (8)	−0.0077 (7)	−0.0012 (7)
C12	0.0486 (10)	0.0415 (9)	0.0377 (9)	−0.0174 (8)	−0.0083 (7)	−0.0019 (7)
C13	0.0512 (10)	0.0381 (9)	0.0462 (10)	−0.0161 (8)	−0.0066 (8)	−0.0008 (7)
N13	0.0659 (10)	0.0370 (8)	0.0624 (10)	−0.0180 (7)	−0.0122 (8)	0.0017 (7)
O14	0.1120 (13)	0.0471 (8)	0.0688 (10)	−0.0313 (8)	−0.0292 (9)	−0.0051 (7)
O15	0.1339 (15)	0.0511 (9)	0.0784 (11)	−0.0470 (10)	−0.0178 (10)	0.0146 (8)
C14	0.0723 (13)	0.0535 (11)	0.0439 (10)	−0.0271 (10)	−0.0127 (9)	0.0088 (8)
C15	0.0845 (15)	0.0690 (13)	0.0398 (10)	−0.0371 (11)	−0.0171 (10)	0.0012 (9)
C16	0.0724 (13)	0.0543 (11)	0.0467 (10)	−0.0325 (10)	−0.0109 (9)	−0.0047 (8)
C21	0.0509 (10)	0.0436 (9)	0.0493 (10)	−0.0228 (8)	−0.0130 (8)	−0.0018 (8)
C22	0.0623 (12)	0.0505 (11)	0.0479 (10)	−0.0202 (9)	−0.0164 (9)	0.0015 (8)
C23	0.0650 (12)	0.0672 (13)	0.0484 (11)	−0.0253 (11)	−0.0078 (9)	−0.0065 (9)
C24	0.0579 (12)	0.0596 (12)	0.0693 (14)	−0.0199 (10)	−0.0076 (10)	−0.0179 (10)
C25	0.0702 (13)	0.0401 (10)	0.0814 (15)	−0.0167 (10)	−0.0171 (11)	−0.0040 (10)
C26	0.0683 (12)	0.0432 (10)	0.0593 (12)	−0.0253 (9)	−0.0111 (9)	0.0034 (8)

### Geometric parameters (Å, °)

**Table:**

C1—N2	1.257 (2)	C13—C14	1.381 (3)
C1—C11	1.478 (2)	C13—N13	1.470 (2)
C1—H1	0.9300	N13—O14	1.218 (2)
N2—C3	1.473 (2)	N13—O15	1.227 (2)
C3—C4	1.509 (2)	C14—C15	1.373 (3)
C3—H3A	0.9700	C14—H14	0.9300
C3—H3B	0.9700	C15—C16	1.383 (3)
C4—C5	1.339 (2)	C15—H15	0.9300
C4—C6	1.481 (3)	C16—H16	0.9300
C5—C21	1.473 (2)	C21—C22	1.389 (3)
C5—H5	0.9300	C21—C26	1.395 (3)
C6—O7	1.203 (2)	C22—C23	1.373 (3)
C6—O8	1.342 (2)	C22—H22	0.9300
O8—C9	1.445 (2)	C23—C24	1.376 (3)
C9—H9A	0.9600	C23—H23	0.9300
C9—H9B	0.9600	C24—C25	1.375 (3)
C9—H9C	0.9600	C24—H24	0.9300
C11—C16	1.387 (2)	C25—C26	1.382 (3)
C11—C12	1.388 (2)	C25—H25	0.9300
C12—C13	1.380 (2)	C26—H26	0.9300
C12—H12	0.9300		
			
N2—C1—C11	122.48 (16)	C12—C13—N13	118.04 (16)
N2—C1—H1	118.8	C14—C13—N13	119.12 (16)
C11—C1—H1	118.8	O14—N13—O15	122.86 (17)
C1—N2—C3	116.16 (15)	O14—N13—C13	118.70 (15)
N2—C3—C4	113.24 (14)	O15—N13—C13	118.44 (17)
N2—C3—H3A	108.9	C15—C14—C13	118.01 (17)
C4—C3—H3A	108.9	C15—C14—H14	121.0
N2—C3—H3B	108.9	C13—C14—H14	121.0
C4—C3—H3B	108.9	C14—C15—C16	120.37 (18)
H3A—C3—H3B	107.7	C14—C15—H15	119.8
C5—C4—C6	121.26 (17)	C16—C15—H15	119.8
C5—C4—C3	124.23 (17)	C15—C16—C11	121.17 (18)
C6—C4—C3	114.43 (16)	C15—C16—H16	119.4
C4—C5—C21	127.15 (17)	C11—C16—H16	119.4
C4—C5—H5	116.4	C22—C21—C26	118.14 (17)
C21—C5—H5	116.4	C22—C21—C5	120.03 (16)
O7—C6—O8	122.52 (18)	C26—C21—C5	121.82 (16)
O7—C6—C4	123.31 (18)	C23—C22—C21	121.17 (18)
O8—C6—C4	114.17 (16)	C23—C22—H22	119.4
C6—O8—C9	115.78 (16)	C21—C22—H22	119.4
O8—C9—H9A	109.5	C22—C23—C24	120.28 (19)
O8—C9—H9B	109.5	C22—C23—H23	119.9
H9A—C9—H9B	109.5	C24—C23—H23	119.9
O8—C9—H9C	109.5	C25—C24—C23	119.5 (2)
H9A—C9—H9C	109.5	C25—C24—H24	120.3
H9B—C9—H9C	109.5	C23—C24—H24	120.3
C16—C11—C12	118.96 (16)	C24—C25—C26	120.70 (19)
C16—C11—C1	120.11 (16)	C24—C25—H25	119.7
C12—C11—C1	120.93 (15)	C26—C25—H25	119.7
C13—C12—C11	118.65 (16)	C25—C26—C21	120.21 (19)
C13—C12—H12	120.7	C25—C26—H26	119.9
C11—C12—H12	120.7	C21—C26—H26	119.9
C12—C13—C14	122.84 (17)		
			
C11—C1—N2—C3	−175.74 (16)	C14—C13—N13—O14	−170.73 (18)
C1—N2—C3—C4	−121.34 (18)	C12—C13—N13—O15	−171.67 (17)
N2—C3—C4—C5	−101.1 (2)	C14—C13—N13—O15	8.4 (3)
N2—C3—C4—C6	82.07 (19)	C12—C13—C14—C15	−0.2 (3)
C6—C4—C5—C21	−175.96 (15)	N13—C13—C14—C15	179.74 (18)
C3—C4—C5—C21	7.5 (3)	C13—C14—C15—C16	0.1 (3)
C5—C4—C6—O7	175.67 (18)	C14—C15—C16—C11	0.0 (3)
C3—C4—C6—O7	−7.4 (2)	C12—C11—C16—C15	0.0 (3)
C5—C4—C6—O8	−4.2 (2)	C1—C11—C16—C15	−179.85 (18)
C3—C4—C6—O8	172.71 (14)	C4—C5—C21—C22	−138.14 (19)
O7—C6—O8—C9	0.2 (3)	C4—C5—C21—C26	42.9 (3)
C4—C6—O8—C9	−179.94 (16)	C26—C21—C22—C23	−1.1 (3)
N2—C1—C11—C16	171.68 (18)	C5—C21—C22—C23	179.90 (17)
N2—C1—C11—C12	−8.2 (3)	C21—C22—C23—C24	1.2 (3)
C16—C11—C12—C13	−0.1 (3)	C22—C23—C24—C25	−0.5 (3)
C1—C11—C12—C13	179.71 (16)	C23—C24—C25—C26	−0.3 (3)
C11—C12—C13—C14	0.3 (3)	C24—C25—C26—C21	0.4 (3)
C11—C12—C13—N13	−179.70 (15)	C22—C21—C26—C25	0.3 (3)
C12—C13—N13—O14	9.2 (3)	C5—C21—C26—C25	179.29 (17)

### Hydrogen-bond geometry (Å, °)

**Table:**

*D*—H···*A*	*D*—H	H···*A*	*D*···*A*	*D*—H···*A*
C23—H23···O15^i^	0.93	2.55	3.307 (3)	139

Symmetry codes: (i) *x*, *y*+1, *z*−1.
